# Beyond automation: How IT professionals utilize the invisible AI arm?

**DOI:** 10.1371/journal.pone.0343114

**Published:** 2026-02-17

**Authors:** Ljupcho Efremov, Ivan Petrov, Irena Nikolovska

**Affiliations:** 1 Liberal Arts Department, American University of the Middle East, Egaila, Kuwait; 2 University of American College, Skopje, North Macedonia; 3 Department of Management, College of Business, University of Doha for Science and Technology, Doha, Qatar; Tata Consultancy Services Ltd, UNITED STATES OF AMERICA

## Abstract

Artificial intelligence (AI) is transforming task optimization across many occupations, particularly within the IT sector. Understanding how IT professionals use AI is crucial for establishing benchmarks and informing best practices. This mixed‑methods study combined two in‑depth interviews with AI‑using IT professionals and a survey of 50 IT professionals in North Macedonia. The survey examined which AI tools are used, for which tasks, and how often they are used in daily work. The results indicate that AI is integrated into multiple aspects of IT work, with task verification (coding assistance and technical documentation) as a common application. ChatGPT emerged as the preferred AI tool, frequently used for documentation and coding, and many IT managers reported using AI tools on a daily basis. These findings suggest that IT professionals go beyond simple automation by routinely embedding AI tools like ChatGPT into their workflows. The insights provide practical guidance for IT professionals and HR departments on how to plan tasks, design AI training, and support productivity and well‑being at work.

## 1. Introduction

Artificial Intelligence (AI) originated in the mid-1950s as noted by Venkatesh [1]. Despite its early potential, progress stalled for several reasons, including technological constraints related to data processing capabilities, management of diverse data types, and the approximation of human thought. However, significant technological advancements have been crucial in the resurgence of AI tools, which now address the limitations of the past. The expansion of AI tools and their potential organizational benefits are unprecedented. Consequently, organizations are actively investing in, deploying, and utilizing AI tools across various operational areas to capitalize on its advantages, establish a competitive edge, and improve overall performance. Approximately twelve years ago, the study of Bravo et al. [2] noted that Artificial Intelligence (AI) has served as a development tool for over two decades, yielding solutions across various Exploration and Production (E&P) sectors, including virtual sensing, production control and optimization, forecasting, and simulation. However, the study highlights that AI applications have not yet become standard industry solutions, with most implementations remaining as case studies and pilot projects. Their work aims to help production and asset management staff understand how AI tools can bring more value and support better decisions. The study also gives guidance on how to choose the right AI applications for different problems. In light of this study’s findings, it is important to note the conclusion of Efremov and Pruthi [3], who emphasized that technological innovations have transformed societies and impacted lives worldwide.

AI has held a place of fascination and speculation for decades, capturing the imaginations of many, particularly through cultural touchstones like the film Terminator in which AI was portrayed in heroic and villainous roles [4], as noted by Kaiser and Meda. These authors highlight the significant progress since the era of Alan Turing, noting the current integration of AI systems into our everyday lives, describing this AI evolution as revolutionary. While AI has developed over time, the past year has seen an exceptional acceleration in its growth and application, signifying a crucial turning point in technological history. The landscape of software development, testing, and IT operations is changing at an unprecedented rate, with market forces, more so than technology itself, dictating this speed. The digital age has compelled organizations to accelerate innovation, deliver intricate solutions, and uphold stringent quality standards, all while adapting to a constantly shifting technological environment. A new trend is emerging as the pressure to optimize processes and shorten time-to-market grows stronger: the incorporation of AI into the fundamental practices of software engineering. The study of Kučević et al. [5] highlights the significant advantages AI offers organizations, including boosted productivity, lower costs, automated processes, and the creation of innovative services and business models. Nevertheless, many organizations, especially resource-constrained small and medium-sized enterprises (SMEs), encounter hurdles in effectively implementing AI due to issues like a lack of specialized knowledge or poor data quality. To tackle these obstacles, these authors develop a comprehensive framework for pinpointing and consolidating the key elements for successful AI adoption. Employing a design science research (DSR) methodology, their study integrates findings from a systematic literature review with insights gathered from expert interviews across diverse sectors. The result is a streamlined AI adoption framework intended to facilitate AI uptake by grouping success factors into key areas: Strategy and Planning, AI Expertise and Support, Data Considerations, Infrastructure and Resources, Market and Competition, Ethical and Legal, and Implementation and Integration. Each area includes interconnected elements, which are classified according to opinion of professionals of prevalence in literature. The aim of this framework is to serve as a kick-off point for small and medium enterprises in order to achieve easier adoption of AI. It underscores the importance of critical success factors such as defining an AI strategy, establishing robust data structures, cultivating an innovative organizational culture, and addressing ethical implications. By focusing on these factors, organizations can reduce risks and improve AI integration into daily work.

Artificial Intelligence has enabled innovative opportunities for assessment creation across businesses, industries, communities, and society as noted by Ijaz et al. [6]. Their research indicates the critical role of technology in various facets of life, leading to its widespread integration within numerous enterprises and sectors. The potential applications of AI are extensive. Specifically, the authors discuss the applications of artificial intelligence (AI) in data science, big data analytics, cybersecurity, Geographic Information Systems (GIS), and nanotechnology. The authors highlight the following AI applications which are relevant for the current study: Data Science (predictive analytics, natural language processing for text analysis, recommender systems, anomaly detection) Big Data Analytics (real-time stream processing, automated data preparation, cluster analysis for segmentation, predictive maintenance, fraud detection) and Cybersecurity (intrusion detection systems, threat intelligence and analysis, behavior-based authentication, malware detection and prevention, security information and event management).

Ayidiya [7] conducted interviews with ten IT professionals possessing a minimum of five years of professional experience in Development Security and Opeations (DevSecOps) and having managed teams of at least three DevSecOps specialists. A prerequisite for participation was their experience as cybersecurity professionals who had either implemented or were in the process of implementing AI within their pipeline. The selection of these experienced participants was a crucial step in the research, ensuring that their direct involvement with the research topic would provide valuable insights. The author writes that the subsequent stage of innovation involves developing automation and incorporating advanced security features, such as artificial intelligence (AI), into the process. Enhanced automation has the potential to decrease the ongoing manual workload of information security professionals, resulting in a more streamlined system. Given that ideal software development environments are frequently unattainable in real-world scenarios, information security professionals encounter various obstacles. Consequently, this study aimed to explore the perceived knowledge gap and determine if it hinders information security professionals from effectively integrating AI into the development, security and operations pipeline. In the DevSecOps context, Ayidiya found that experienced cybersecurity professionals recognize the potential of AI and automation to reduce manual workload, but also report barriers such as limited knowledge, high costs, and shortage of skilled experts. These perceptions show that even in advanced IT environments, AI integration is not straightforward (a culture of security awareness and technical training alone would not be adequate to shift the perceptions of cybersecurity experts).

The paper by Ramos [8] examines the wide-ranging effects of AI adoption on today’s business landscape, specifically addressing the economic ramifications and the technological advancements driving this transformation. Economically, the increasing integration of AI technologies is reshaping traditional business models by altering the dynamics of production, distribution, and consumption. Automation, a central feature of AI, is poised to enhance operational efficiency across various industries, leading to improved productivity. Furthermore, the AI-powered revolution in decision-making, leveraging vast amounts of real-time data, allows businesses to extract valuable insights and make precise strategic adjustments. This capability facilitates streamlined operations, the development of novel products, and the attainment of a competitive edge, ultimately fueling economic growth and market expansion. More specifically, Wirawan and Oktavia [9] point out that software developers can benefit from using ChatGPT. Developers often face issues like complex code or algorithms that are hard to understand, and problems in the code that are difficult to fix. This study explores the factors that affect software developers’ decisions to use ChatGPT for programming information. Using a model called UTAUT, the researchers surveyed 399 software developers and analyzed the data with a method called PLS-SEM. The results show that Performance Expectancy, Effort Expectancy, Trust, Perceived Risk, and Experience all significantly influence whether developers intend to use ChatGPT. Also, their intention to use ChatGPT greatly affects their actual adoption of it. Cheuk et al. [10] have emphasized the necessity for contemporary IT professionals to adopt software development strategies that prioritize both speed and quality. They highlight the growing importance of integrating security measures into the design phase to facilitate the detection, resistance, reaction, and recovery from cyberattacks. The authors posit that near-complete automation represents the future of software development. A more immediate objective is to foster and improve automation across the software delivery pipeline to minimize errors and downtime. Their paper examines current automation technologies within the software security field to assess their efficacy and to propose avenues for future research and insights. Chubb et al. [11] interviewed leading scholars to explore the potential impact of AI on research practice and culture. Through deductive, thematic analysis, they identified issues currently affecting academics and universities. Their interviewees highlighted both positive and negative consequences of AI, considering both collective and individual applications. AI was seen as beneficial for information gathering and specific, narrow tasks, as well as in supporting research impact and interdisciplinarity. However, the scholars cautioned that using AI merely to “speed up” processes to meet bureaucratic and metric-driven demands could exacerbate negative aspects of academic culture. They emphasized that the integration of AI in research should serve to augment, not supplant, human creativity. In this line is the study of Garcia et al. [12] who argue that AI, especially with the emergence of natural language processing tools like ChatGPT, has demonstrated significant relevance in streamlining routines, aligning with theoretical predictions. The authors argue that the potential of AI to enhance daily corporate operations has dramatically increased. Current technology is exerting growing influence in management, offering numerous advantages such as time efficiency and the rapid, effective delivery of pertinent information. The authors conducted a qualitative study with AI users from different companies in order to comprehend actual AI usage. Their analysis revealed that users consider AI a valuable asset for supporting decision-making and problem-solving. Despite these positive views, concerns regarding the accuracy and reliability of AI persist. Some users indicated a need to verify AI-generated information before full reliance, suggesting a degree of skepticism or caution about its precision and trustworthiness. On the other hand, the findings strongly suggest high confidence and acceptance, and indicate substantial receptiveness among examined professionals. The responses reveal a widespread popularization of AI usage across various sectors, notably among individuals outside the IT domain and from diverse fields, including participants from the meat industry, advertising agencies, and veterinary clinic networks. The results of the above-mentioned studies can be explained by Technology Acceptance Model (TAM), which explains technology adoption mainly through perceived usefulness and perceived ease of use. According to Ibrahim et al [13], the model was developed by Fred Davis in the nineties of 20th century. TAM model explains why people accept or reject a new technology based mainly on two beliefs: perceived usefulness (how much it helps them do their job better) and perceived ease of use (how easy it is to use). These beliefs shape the user’s attitude and intention to use the system, which then predicts actual usage.

The general expectation is that IT professionals are leaders in usage of AI at work and therefore they were selected as a main target group for the research. However, the key questions concern how AI is used in daily work. AI is a relatively new field, and there is limited research on how IT professionals concretely use AI tools in their daily work, especially in North Macedonia. The current study fills this gap by providing initial knowledge and baseline on AI usage by this specific group. The aim of the research is to identify how IT professionals use AI tools. More specifically, the research delves into which AI tools are used, what kind of tasks AI is used for and how often AI tools are used in their everyday routine. The research question stated in the title is how IT professionals integrate AI tools into daily tasks? Additionally, there were two specific hypotheses which were tested. The first hypothesis is about the prevalence of ChatGPT among the tools, while the second hypothesis was that IT employees would use AI tools for coding assistance more than 50%. The hypotheses are derived from expectations that ChatGPT is the first and most known AI tool, while the expectation for coding was posed due to possibility of a fast and accurate double-check of code.

## 2. Methodology

The research utilizes a combination of qualitative and quantitative methods. The expectation is that IT professionals lead the usage of AI in their work, but the key point is how they use AI. During the initial stage, in-depth interviews were conducted with 2 IT managers who have used AI in their daily work. The purpose of the interviews was to examine current usage and potential topics which would later be included in the survey as a second stage. The mixed-methods design started with semi-structured interviews of two IT managers (15–20 minutes each) to explore AI usage patterns and identify main themes for later survey. These themes were refined into four validated questions about AI tool usage, specific tools (for example ChatGPT and some of the most used other tools), task types such as coding, frequency of use, plus demographic section. From the initial interviews, the key concepts were extracted through iterative mapping of repeated rounds between researchers where raw themes were linked to draft questions, checked alignment and gaps, revised based on feedback from the three researchers, and repeated until content validity was good. Reflexive notes and peer debriefing among the research team helped to ensure trustworthiness. Items were piloted with small group of five IT professionals (not in final sample), refined for face and content validity after review by two IT experts (high agreement rates), and piloting confirmed clear wording and consistent responses. Afterwards, using the abovementioned input from both interviews, a questionnaire was created. The questionnaire included four AI specific questions: usage of AI for work, usage of specific AI tools, what kind of tasks AI is used for and how often AI-powered tools are used. Additionally, a section on demographics was added. Participants were given instructions that they can withdraw from the study, that the study is anonymous and results will be used for research purposes only. Fieldwork for the study was conducted in March and April 2025 using Google Forms. A snowball sampling method was used. A total of 50 IT managers were included in the survey. The sample consisted mostly of IT managers with multiple roles in IT field (28%), followed by software developers (24%). The gender distribution shows that 80% of the IT professionals were male and 20% of them were females. The results are presented in percentages and Chi-square and Binomial Test for a Single Proportion were performed. Both hypotheses (the first hypothesis about the prevalence of ChatGPT among the tools and the second about usage for coding assistance more than expected) were tested with binomial test. Given the sample size (n = 50) and the structure of the questions (mostly categorical like tool choice or frequency groups), appropriate tests were Chi-square for checking associations between variables and binomial test for single proportion hypothesis (if ChatGPT use is over 50% as expected). These basic tests match the data type and size and are appropriate for this initial and exploratory study. The inferential analyses (Chi‑square and binomial tests) were used in an exploratory way, with the understanding that statistical power is limited and non‑significant results may reflect insufficient power rather than the absence of an effect. The analysis remains mostly descriptive, which fits the exploratory aim of the study and the initial expectations. It is one of the first studies of its kind that seeks to map AI usage patterns among IT professionals in North Macedonia.

## 3. Results

The results are presented in the tables below. The results show that all of the surveyed participants (100%) use AI tools in their daily work. The first presented question is about frequency of usage (Table 1).

**Table 1:** Presentation of results regarding frequency of usage of AI tools.

Options	At least once a day	Once a week	Once a month or les
Q1. How often do you use AI-powered tools in your daily work routine?	56.0	36.0	8.0

**Source:** author’s own study

The chi-square test yielded a statistically significant result (χ2 = 17.435, df = 2, p < 0.001), indicating that the frequency of AI-powered tool usage in daily work routines is not equally distributed across the three options. Specifically, the observed frequency of using AI tools at least once a day was significantly higher than expected under the assumption of equal proportions, while usage once a month or less was significantly lower.

The next table (Table 2) presents results about usage of specific AI tools. The expectation is that ChatGPT would be more frequently used than other tools.

**Table 2:** Presentation of results regarding most frequent usage of specific AI tools.

Q2. Which of the following AI tools do you use most frequently (given in alphabetical order in the question)? (Select all that apply)?	Percentage (decreasing order)
ChatGPT (OpenAI)	90
Copilot (Microsoft)	32
DeepSeek AI	30
Perplexity AI	22
Gemini (Google DeepMind)	16
Claude (Anthropic)	14
Bing Chat (Microsoft)	10
Llama (Meta)	10
Siri (Apple)	10
Other	4
Mistral AI	2
Watson (IBM)	2

**Source:** author’s own study

The expectation was that ChatGPT as a first and most known tool would have the highest percentage of usage among IT managers. The significant binomial test (n = 50, x = 45, p < 0.001) demonstrates that the observed proportion of ChatGPT usage (90%) is significantly higher than a hypothesized proportion of 50%. This indicates that ChatGPT is used by a significantly larger proportion of the sample than would be expected if it were used by only half the respondents. The results strongly support the expectation that ChatGPT is the most frequently used AI tool among the respondents. With an estimated 90% of the sample (45 out of 50 individuals) selecting it, ChatGPT’s usage significantly surpasses that of all other listed tools. This dominance is evident when compared to the usage rates of Copilot (32%), DeepSeek AI (30%), and the considerably lower percentages for the remaining options. The data clearly indicates that ChatGPT is the most prevalent AI tool in this context.

The next table (Table 3) is about usage of AI tools for specific tasks. The expectation is that coding assistance would be used more than expected. The descriptive analysis reveals that generating documentation or technical writing (62%) and coding assistance (58%) are the most frequently reported uses of AI. The results which show the types of tasks that are used by IT professionals are shown in the table below. While the initial expectation focused on coding assistance being the primary use, the data indicates high usage for both these tasks. To statistically examine the usage of coding assistance, a binomial test was conducted to compare the observed proportion (58%) to a hypothesized proportion of 50%.

**Table 3:** Presentation of results regarding specific tasks for which AI is being used.

Q3. Please indicate for what kind of tasks do you use AI? (Select all that apply)?	Percentage
Generating documentation or technical writing	62
Coding assistance	58
Data analysis	44
Code review or bug detection	36
Automating repetitive tasks	34
Predictive analytics	30
Cyber-security tasks	14
Other	8

**Source:** author’s own study

The binomial test yielded a p-value of 0.1841 (n = 50, x = 29). Since this p-value is greater than the significance level of 0.05, we fail to reject the null hypothesis. Therefore, based on this test, there is not enough statistical evidence to conclude that the proportion of AI use for coding assistance is significantly greater than 50%. However, the observed high percentages for both coding assistance and technical writing suggest that AI is prominently utilized for these technical tasks within this sample, even if the binomial test against a 50% benchmark for coding assistance alone did not reach statistical significance at the 0.05 level. Further investigation with different benchmarks or comparisons between the usage rates of different tasks might discover additional insights.

## 4. Discussion

AI tools are universally adopted among surveyed IT managers, and daily usage is the norm. ChatGPT is by far the most frequently used tool, and AI is mainly applied for documentation and coding assistance, showing broad integration of AI into daily technical tasks. Additionally, the frequency of using AI tools is particularly notable: the proportion of IT managers using AI at least once a day is significantly higher than those using it weekly or monthly. This pattern reflects a strong and routine reliance on AI in daily workflows, a trend that aligns with broader global data showing rapid and deepening integration of AI tools – especially ChatGPT – across office environments worldwide. A specific statistic on the exact proportion of IT managers using AI daily isn’t explicitly available in the literature and therefore this study provides meaningful contribution to the debate about the increasing adoption of AI tools by managers, including IT managers, in their daily workflows. The strong adoption of AI by IT managers is also coherent with Sahay and Kaur [14] who showed that automation, digitization, and artificial intelligence (AI) lead to much better results in organizations. They found that AI allows managers to get large amounts of employee performance data in real-time. This data can be used for evaluations that are unbiased and fair, which can improve how well the organization performs. The first hypothesis about expectation regarding high prevalence of ChatGPT is confirmed. The results are in line with the study of Jo and Park [15] who examined what makes workers use ChatGPT and what the results of that use are. They studied 351 people from different work areas, aged 20–40. The study found that when workers think ChatGPT is smart and can learn on its own, they are more likely to get information support and gain knowledge from it. This, in turn, makes them see ChatGPT as useful. If workers see ChatGPT as useful, they intend to use it. And if they intend to use it, they are more likely to actually use it. Similarly, Ibrahim et al. [13] found that perceived usefulness and a positive AI mindset are key drivers of AI adoption, consistent with the Technology Acceptance Model (TAM). The results of current study fit this pattern: ChatGPT’s dominance in the sample suggests that IT managers perceive it as both useful and easy to use, and therefore integrate it into daily tasks such as coding assistance and technical documentation. They rely on it as a practical tool to work faster, double-check their ideas, and reduce routine workload. Additionally, the obtained results from the study show that AI is mostly used for coding and technical writing. This focus on documentation and coding assistance is consistent with Bringula [16] who showed that ChatGPT supports teachers and students in generating teaching materials and programming code. However, the study identified some downsides, including ChatGPT’s tendency to provide incorrect sources, create repetitive content, and the risk of it being misused. Although the second hypothesis is not confirmed, more than half of the IT professionals use AI for coding. This hypothesis might have been confirmed if only software developers were included in the sample. The results of the study provide valuable contribution to understanding of AI adoption among IT professionals.

The limitations of this study are mainly connected to the snowball sampling method, small sample size of 50 IT managers, and focus only on North Macedonia, which limits generalizability to other countries or regions. Snowball sampling was chosen because IT professionals are hard to reach directly, but it may create bias toward more connected respondents and a less diverse group. Also, all participants were based in North Macedonia, so the findings reflect local regional context and may not transfer directly to other regions, such as Western Europe or the USA, where AI tools or work culture may differ. The statistical power of this study is limited because of the relatively small sample size (n = 50). This means that some real effects may not be detected by the inferential tests. Therefore, the results of the Chi‑square and binomial tests should be interpreted with caution and mainly as exploratory indications rather than strong confirmatory evidence. To enhance the generalizability of the findings, future studies should include larger and more representative samples. Furthermore, future research should compare usage rates across different tasks and AI tools to provide a more comprehensive understanding of how and why specific tools and tasks are prioritized by IT managers. This would help identify broader trends and inform best practices for AI adoption in the workplace. Additionally, in-depth interviews should be conducted to gain deeper insights into the reasons behind the selection of specific AI tools and the motivations for using AI in particular tasks. These qualitative findings could complement the survey data, helping organizations better understand user preferences, challenges, and the practical impact of AI integration in daily IT management.

Overall, the findings confirm that AI tools, especially ChatGPT, have become a routine part of IT management work. The final conclusion is that IT professionals utilize AI beyond automation by embedding tools like ChatGPT into daily tasks such as generating documentation and coding assistance, reflecting a routine reliance on AI-enhanced workflows. In this way, AI functions as an “invisible arm” that quietly supports and extends IT professionals’ capabilities behind the scenes. Organizations can use these insights to prioritize AI training, invest in the most widely adopted tools, and focus on integrating AI solutions for documentation, coding, and data analysis tasks. Understanding these usage patterns can help IT leaders make informed decisions about resource allocation, workflow optimization, and future technology adoption.
